# Use of Sugar Dispensers at Lower Density Can Decrease Mealybug (Hemiptera: Pseudococcidae) Infestation in Vineyards by Disrupting Ants

**DOI:** 10.3390/insects16050468

**Published:** 2025-04-29

**Authors:** Giovanni Burgio, Serena Magagnoli, Luca Casoli, Marco Profeta, Donato Antonio Grasso, Enrico Schifani, Daniele Giannetti, Martina Parrilli

**Affiliations:** 1 Department of Agricultural and Food Sciences, University of Bologna, 40127 Bologna, Italy; giovanni.burgio@unibo.it (G.B.); parrillimartina@gmail.com (M.P.); 2 Consorzio Fitosanitario Provinciale di Reggio Emilia, 42124 Reggio Emilia, Italy; luca.casoli@regione.emilia-romagna.it (L.C.); marco.profeta@regione.emilia-romagna.it (M.P.); 3 Department of Chemical Life Sciences and Environmental Sustainability, University of Parma, 43124 Parma, Italy; donatoantonio.grasso@unipr.it (D.A.G.); enrico.schifani@unipr.it (E.S.); daniele.giannetti@unipr.it (D.G.)

**Keywords:** *Pseudococcus comstocki*, *Anagyrus vladimiri*, *Cryptolaemus mountrouzieri*, Formicidae, biological control

## Abstract

Vineyard mealybugs (Hemiptera: Pseudococcidae) are economic pests in vineyards, demanding integrated control strategies. Several ant species can facilitate mealybug infestation by protecting them from natural enemies in a mutualistic relationship known as trophobiosis. In the frame of an ant management system, the provision of sugary liquid has proved worldwide to improve mealybug control. In the present study, a field trial was carried out within an important vineyard cultivation area of northern Italy with the aim of testing a lower density (80/ha) of sugar dispenser to facilitate the practicality of this method. The sugar dispensers tested, along with predators and parasitoid release, were effective in reducing mealybug infestations by 22% and resulted in a double increase in larval density of the predator *Cryptolaemus mountrouzieri* Mulsant. Mealybug parasitism was in general high, but it was not improved by sugar dispensers at this density. Our field validation confirms the importance of ants in mealybug infestation dynamics, and the benefits of ant management in the context of integrated strategies against mealybugs.

## Simple Summary

Mealybugs (Hemiptera: Pseudococcidae) are an important economic pest in vineyards. The use of sugar dispensers has been proposed as a practical method to reduce mealybug infestations. In this study, we found they increased the predator *Cryptolaemus mountrouzieri*; however, mealybug parasitism was in general high and not affected by sugar dispensers. Additionally, higher mealybug infestations were recorded in vineyards with a higher presence of *Lasius* ants, which are characterized by a high tendency to feed on hemipteran honeydew. This experiment confirms that ant management should be implemented for integrated control of mealybugs.

## 1. Introduction

Vineyard mealybugs (Hemiptera: Pseudococcidae) have recently increased their harmfulness, causing significant economic losses [1]. In an Italian scenario, two main mealybug species are considered key pests: the vine mealybug, *Planococcus ficus* (Signoret), and the Comstock mealybug, *Pseudococcus comstocki* (Kuwana) (Hemiptera: Pseudococcidae) [2,3]. The first record of the Comstock mealybug in Western Europe (Italy and France) dates back to 2004 [4], and since 2018, this species has caused serious damage to grapevines in Italy, especially in northern regions [5,6,7].

Several factors affect mealybug infestations and the related damage they cause, including nitrogen fertilization, pesticide use, the presence of natural enemies, and ant abundance and activity, all of which can contribute to pest outbreaks [8,9,10]. Broad-spectrum pesticides alone do not provide adequate control, and sustainable methods such as mating disruption [3] and biological control are needed [7,11,12]. For these reasons, an integrated approach involving multiple methods has been evoked to manage and mitigate mealybug infestations in vineyards.

Ants play a significant role in providing ecosystem services in terrestrial environments, including agroecosystems, where they contribute to seed dispersal, biological control, soil formation, nutrient retention, and, in some cases, pollination [13,14]. In particular, ants have been reported to provide biological control of pests in various cultivated systems and areas [15,16,17,18,19,20]. In addition to this beneficial role, ants can also be harmful in agriculture. Some species (i.e., leafcutter ants and harvester ants), for example, are crop pests in some countries [21,22]. Moreover, many ant species have developed a mutualistic relationship, known as trophobiosis, with many sap-sucking species, including mealybugs, due to their honeydew-consuming habit. This relationship can be harmful as ants may facilitate mealybug dispersal, enhance colony hygiene, and protect mealybugs from natural enemies [11]. The mechanisms through which ants benefit their hemipteran partners are variable, depending on the specific ant species and the ecological context [9,23]. Due to the high frequency of trophobiotic interactions, trophobiosis has recently been proposed as a tool for monitoring vine mealybugs infestations, based on ant behavior [13].

A number of management practices have been recently developed to manipulate ant–sap-sucking associations, including barriers for ant exclusion, ant-tolerant predators and parasitoids, toxic baits, and alternative food sources [24]. The latter method involves the provision of sugar through dispenser-feeders (artificial nectaries), which can reduce ant attendance at mealybug colonies and mitigate their reproductive behavior. This method has been applied in some crops (i.e., vineyard crop, citrus, and mango) to reduce mealybug infestations and enhance biological control by altering ant movements and foraging behavior [7,25,26,27].

Apart from sugar and water, other substances such as alkaloids can increase ant loyalty to sugary dispensers, further promoting the disruption of trophobiotic interactions [28]. Alternatively, insecticides incorporated into artificial sugar dispensers can contribute to the control of trophobiotic ants [29], although this may negatively affect non-target ants, pollinators, and natural enemies, thereby reducing the ecosystem services they provide [24]. Nevertheless, the small amount of pesticides and bait delivery system used in this approach result in fewer undesirable effects compared to broad-spectrum insecticide sprays [30,31]. Polyacrylamide hydrogels have also been explored as a promising delivery method for sugar baits and insecticides [32,33,34,35].

The provision of sugary liquids has been shown to potentially manipulate ant activity, as well as enhance the beneficial effects of ants in controlling other phytophagous insects and plant pathogens [20,24]. A very peculiar example of this approach was demonstrated in Argentinian vineyards where the Argentine ant *Linepithema humile* (Mayr) (Hymenoptera: Formicidae), a common invader, was able to defend grapevines against leafcutter ants, with artificial sugar sources boosting this defense service [22].

In light of the above, an ant management system can be an integrative method to mitigate mealybug infestations in crops like vineyard crops [11,22,25,27], and seems particularly suitable for systems where biological control needs to be implemented.

In the Lambrusco wine-producing area of the Emilia-Romagna region (mainly Modena and Reggio-Emilia provinces, northern Italy), mealybugs are considered a serious threat. An area-wide augmentative biological control program has been conducted since 2019 [36], releasing the parasitoid *Anagyrus vladimiri* Triapitsyn (Hymenoptera: Encyrtidae) and the predator *Cryptolaemus mountrouzieri* (Mulsant) (Coleoptera: Coccinellidae). In a previous field study conducted in pilot farms within this cultivation area, the sugar provisioning method was effective in reducing mealybug infestations and enhancing biological control. The dispenser density in this validation was relatively high (120/ha), and the method proved to be labor-intensive [7].

The present study employed a field trial in the same cultivation area, using a lower dispenser density (80/ha) to assess a more practical approach with reduced management and application costs. Particular attention was given to estimating the effects of sugar dispensers on mealybug infestation dynamics and biological control, with an evaluation of the potential efficacy range of the dispenser device.

## 2. Materials and Methods

### 2.1. Field Sampling Sites and Experimental Design

Four vineyards of the Reggio Emilia Province were involved in the experiment (Table 1). Vineyards characterized by historical mealybug infestations caused by *P. comstocki* were selected by means of extension service recommendation and managed according to IPM guidelines of the Emilia-Romagna region.

The trial was carried out in an area of 0.5–1.5 ha inside each vineyard, where two plots were set: SD with sugar dispensers at a density of 80 devices/ha and a control plot (C, without sugar dispensers), following a block design, where vineyards represented the blocks. The plot areas varied between 500 and 2.600 m^2^, proportionally to the size of each vineyard, and the buffer zone between the two plots was at least 30 m.

Sugar dispensers were built and refilled every two weeks with 175 mL of a 25% sucrose aqueous solution, following methodology described in the literature [6]. Moreover, they were positioned within each plot on vine trunks (50 cm above the ground) in mid-May and regularly distributed throughout the SD plot at an average distance of 15–20 m from each other.

### 2.2. Assessment of Mealybug Infestations, Natural Enemies, and Ants

Natural enemies were provided by the biofactory Bioplanet (Cesena, Italy) and introduced to ensure a comparable level of ecosystem services in each vineyard. *Anagyrus vladimiri* was released into selected vineyards at a rate of 1.500 individuals/ha (sex ratio: 60% females). The releases took place in mid-July in two separate events spaced one week apart after *Scaphoideus titanus* Ball. (Hemiptera: Cicadellidae) mandatory treatments.

Starting at the end of July, 300 adults/ha of *C. montrouzieri* (sex ratio: 50% females) were also released, focusing on plants with high mealybug infestation. The release of natural enemies was carried out in both treatments (SD and C) of the vineyards involved in the experiment.

Ant individuals were sampled using a fixed time of 1 min and manually collected fortnightly from the canopy and trunks on which sugar dispensers were set in order to define the ant fauna of the vineyards during the experiment (June–August). Ant species were collected with vials and identified in a laboratory by using specific dichotomous keys available in the literature [37].

During pre-harvest (mid-September), infestation was defined by evaluating the percentage of infested bunches and the number of mealybug individuals per bunch in each plot, using variable samples sizes in relation to the plot area (range of sampled bunches per plot varied between 18 and 40).

The percentage of infested bunches was determined directly in the field through random visual sampling carried out on each vine where sugar dispensers were installed and compared to vines in the control plots. Then, the same bunches visually assessed were collected and moved to a laboratory. In addition to random sampling, systematic collection of infested bunches (N = 10) (hereafter called colonies) was employed from plants on which sugar dispensers were set and in control plots.

The estimation of mealybug density was carried out by counting the number of specimens per bunch, and distinguishing nymphs, adults (females), and mature females with an ovisac. Mealybug density estimation was carried out for both bunches randomly and systematically collected.

The parasitizazion rate was calculated both on random and systematically collected bunches by determining the number of adult parasitized mealybugs out of the total of adults counted. Finally, the larval predator density was estimated only on colony samples.

The efficacy range of sugar dispensers was analyzed for infestation, parasitism, and predator larval density. In detail, both random and infested (or colony) bunch samplings were carried out in plants located 5 m from the vine on which sugar dispensers were located and compared to bunches collected from vines where sugar dispensers were positioned (considered at 0 m of distance).

### 2.3. Statistical Analysis

Partial Least Squares [38], a technique that combines principal component analysis and multiple regression, was used to correlate the relative abundance of ant species to mealybug infestation (mealybugs per bunch) in all vineyards. We selected this multivariate method because it is considered particularly useful to predict dependent variables from a large set of independent variables (or predictors), in our case the mealybug infestation and the ant species, respectively [39]. The aim of Partial Least Squares was to correlate ant species with the severity of mealybug infestation.

Log-linear analysis [40] was used for hypothesis testing, to analyze the frequency of the response variables: **i**. infested bunches and **ii.** parasitism, depending on the categorical variables of treatments (sugar dispenser or control) and vineyards. Log-linear analysis allows for the simultaneous evaluation of multiple interactions among more than two categorical variables in multi-way frequency tables using a method similar to factorial experimental design; *p*-values were included in the text—such as those from partial (P(p)) and marginal (P(m)) association tests—only when a discrepancy between these values was detected.

Discrete counts (mealybugs/bunch and predator larvae/bunch) were analyzed by a generalized linear mixed model (*glmm*) with gamma distribution and Log link function, which included treatments (sugar dispenser or control) as a fixed factor and vineyard as a random factor.

Infestation, parasitism, and larval density of the predator were analyzed by excluding one vineyard due to an insecticide treatment during the sampling activity, which could have likely affected natural enemy activity and abundance. However, data from the fourth vineyard were included in Partial Least Square analysis.

Finally, the error bars in the percentage-based graphs represent the binomial error, while those in the mean-based graphs correspond to the standard error of mean.

Statistical analyses were performed using Statistica version 10 (StatsoftTM, Tulsa, OK, USA) and SAS 9.0.

## 3. Results

### 3.1. Ant Fauna Analysis

**Figure 1:**
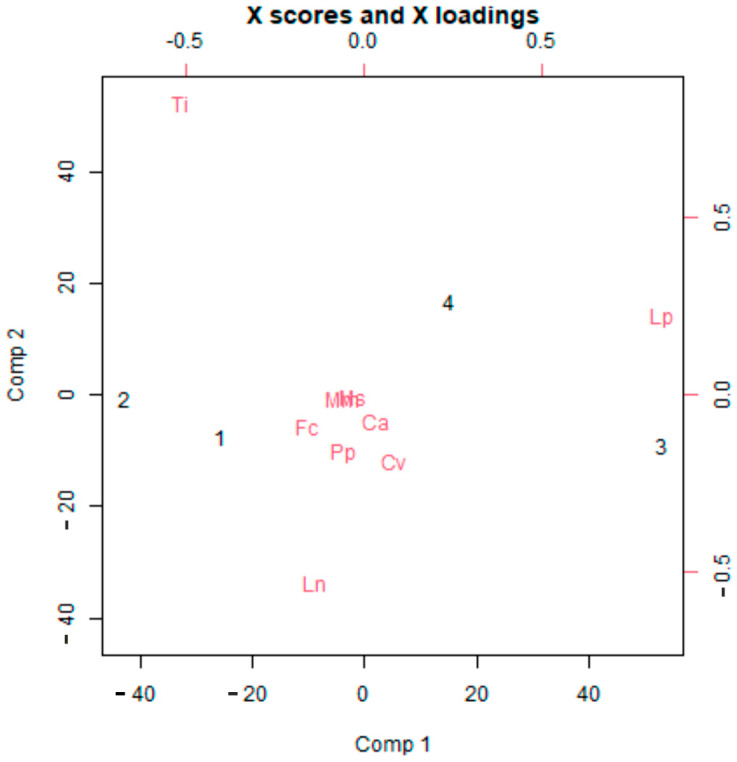
Partial Least Squares calculated for the relative abundance of ant species and the number of mealybugs per bunch in the experimental vineyards (1–4). Ca = *Camponotus aethiops*, Cv = *Camponotus vagus*, Fe = *Formica cunicularia*, Ln = *Lasius niger*, Lp = *Lasius paralienus*, Mm = *Monomorium monomorium*, Ms = *Myrmica sabuleti*, Pp = *Plagiolepis pygmaea*, Ti = *Tetramorium immigrans*. Component 1 (explained variance = 81.5%); Component 2 (explained variance = 6.5%).

Overall, nine ant species were found in the vineyards sampled in the present field trial, with *Lasius paralienus* Fabricius, 1804 (Hymenoptera: Formicidae) (36.7%) and *Tetramorium immigrans* Santschi, 1927 (Hymenoptera: Formicidae) (35.2%) being the dominant species, although ant presence showed some variability among the vineyards. Partial Least Squares analysis showed that most abundant mealybug infestations (vineyards 3 and 4) were correlated with a higher abundance of *L. paralienus* (Lp), which had the highest loading value on the first component (Comp. 1) compared to other ant species. In this analysis, the first component explained that 81.5% of the variance was particularly relevant for correlating ant species with mealybug infestations (Figure 1).

### 3.2. Mealybug Infestation in Sugar Dispenser Versus Control Plots

**Figure 2:**
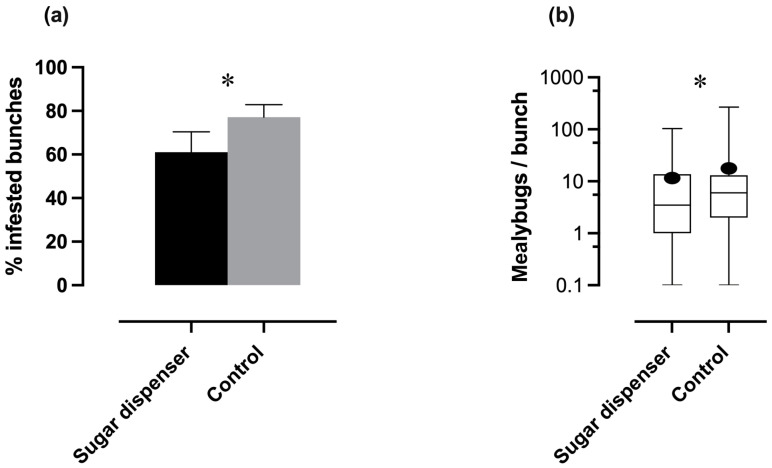
(**a**) Percentage of mealybug-infested bunches of sugar dispenser against control plots (Log-linear analysis, *p* < 0.05). Bars correspond to the binomial error. (**b**) Mealybug density of sugar dispenser versus control plots represented with boxplots. The longitudinal line indicates the median of the distribution; the box represents the interquartile range (from the lower to the upper quartile); and the whiskers extend from the minimum to the maximum values. Black dots represent the mean values (*glmm*, *p* < 0.001). Asterisks indicate significant differences between treatments.

The sugar dispenser (SD) significantly reduced the percentage (%) of mealybug-infested bunches compared to the control (C) (Log-linear analysis, “treatments × infestation rate”, *p* < 0.05, Figure 2a), resulting in an overall normalized infestation reduction of 22%. Additionally, the mean number of mealybugs per bunch showed a significant difference between SD and C plots (*glmm,* AIC = 23.8, Wald test = 19.5, *p* < 0.001, Figure 2b), indicating a reduction in mealybug density due to use of the sugar device.

Mealybug infestations displayed high variability among the vineyards as shown by the significant interaction between “vineyard × infestation rate” in Log-linear analysis (*p* < 0.001) and by the significant “vineyard” effect on mealybug density (*glmm*, AIC = 23.8, Wald test = 2490.3 *p* < 0.05).

### 3.3. Evaluation of the Range of Sugar Dispensers on Mealybug Infestation

**Figure 3:**
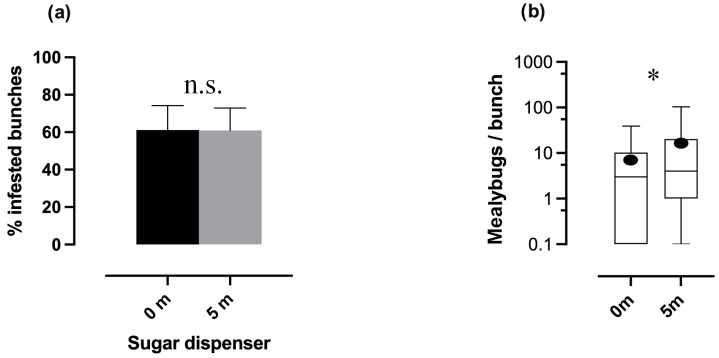
(**a**) Percentage of infested bunches within sugar dispenser plots; the infestations between plants with a sugar dispenser (0 m) and those 5 m from a sugar dispenser (5 m) are compared. (Log-linear analysis, *p* > 0.05). Bars correspond to the binomial error. (**b**) Mealybug density in plants near a sugar dispenser (0 m) and 5 m from a sugar dispenser (5 m). (*glmm*, *p* < 0.001). Boxplot ranges are defined as described in Figure 3b. Asterisks indicate significant differences between distances, while “n.s.” indicates non-significant differences.

Concerning the range of sugar provisioning, no differences were detected in the percentage of infested bunches between plants with dispensers at 0 m (0 m) and those located 5 m away from the dispenser location (5 m) within SD plots (Log-linear analysis, “treatments × infestation rate”, *p* > 0.9, Figure 3a). Nevertheless, the mean number of mealybugs per bunch in SD plots was significantly lower on plants with dispensers (0 m) in comparison with those at 5 m (*glmm,* AIC = 39.2, Wald test = 8.2, *p* < 0.01, Figure 3b).

### 3.4. Effect of Sugar Dispensers on Parasitism Rate and Predator Abundance

**Figure 4:**
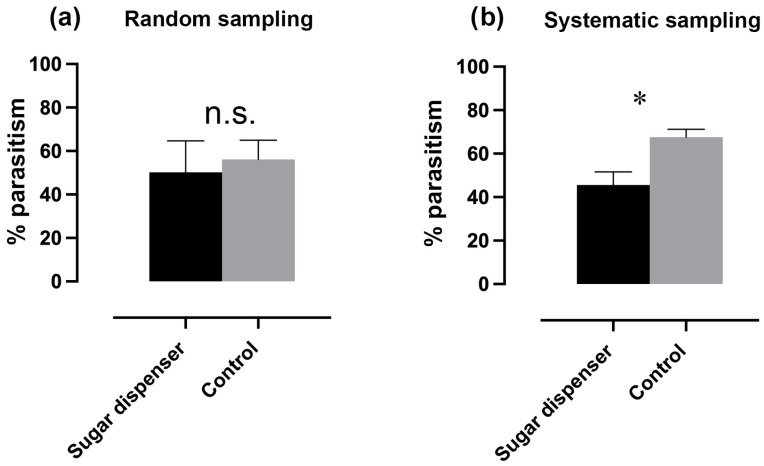
(**a**) Percentage of parasitism estimated by random sampling in SD and C plots. (Log-linear analysis, *p* > 0.05). Bars correspond to the binomial error. (**b**) Percentage of colony parasitism, estimated by systematic sampling in SD and C plots. *p* < 0.01 (Log-linear analysis). Bars correspond to the binomial error. Asterisks indicate significant differences between treatments, while “n.s.” indicates non-significant differences.

Parasitism (%) estimated by random sampling showed no significant difference between SD and C plots (Log-linear analysis, “treatments × *parasitism (random), *p* > 0.05, Figure 4a), denoting a lack of efficacy of the sugar device at the tested parasitism rate as estimated using this sampling method. A discrepancy in the calculated *p* values was observed between partial (P(p) = 0.865) and marginal (P(m) = 0.089) association tests for random parasitism, although both provided statistical values higher than 5%. Mealybug parasitism (%) estimated by random sampling showed significant differences among vineyards (Log-linear analysis, “vineyards × *infestation, *p* < 0.01).

Mealybug parasitism estimated by systematic sampling of infested bunches (referred to as colonies) was in general very high, reaching peaks of about 70%. Colony parasitism (%) was higher in C than in SD plots (Log-linear analysis, “treatments × parasitism (colony)”, *p* < 0.01, Figure 4b), denoting that sugar dispensers at the tested field rate did not increase parasitism. Moreover, colony parasitism (%) displayed high inter-vineyard variability (Log-linear analysis, “vineyards × parasitism (colony)”, *p* < 0.01).

**Figure 5:**
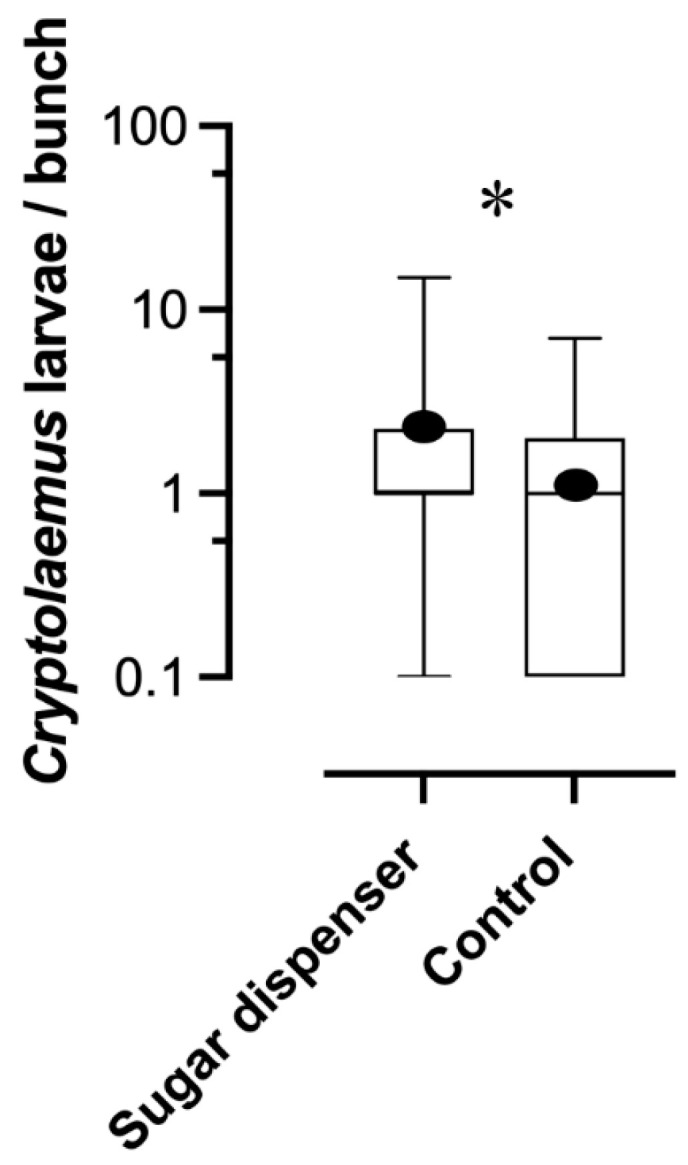
Density of the predator *Cryptolaemus montrouzieri.* Boxplot ranges are defined as described in Figure 3b. (*glmm*, *p* < 0.01). Asterisks indicate significant differences between treatments.

Larval density of the predator *C. montrouzieri* was significantly higher in SD than C plots (*glmm*, AIC = 8.4, Wald test = 22.9, *p* < 0.01) (Figure 5).

### 3.5. Effect of the Distance from Sugar Dispensers on Parasitism and Predator Abundance

**Figure 6:**
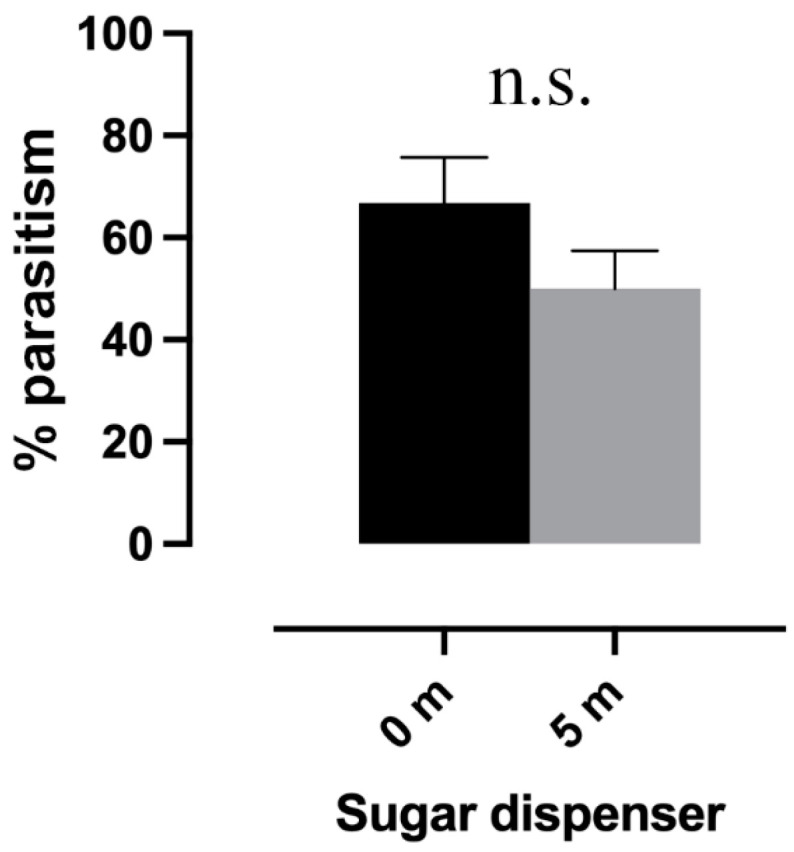
Percentage of parasitism between plants with a sugar dispenser (0 m) and those 5 m from a sugar dispenser (5 m) are compared. (Log-linear analysis, *p* > 0.05). Bars correspond to the binomial error, while “n.s.” indicates non-significant differences between distances.

Mealybug parasitism (%) was higher on plants with dispensers compared to those at a 5 m distance, but this trend was not supported by statistical analysis (P(p) = 0.149, P(m) = 0.267), which did not show significant differences between the treatments (Log-linear analysis, “treatments × parasitism”, *p* > 0.05, Figure 6).

Sampling of the predator larval density on plants with a SD versus those 5 m away did not provide enough data to support statistical analysis as the abundance of predator larvae was very low.

## 4. Discussion

In accordance with a previous study [7], this paper can contribute to the development of an integrated and sustainable strategy for pest control that utilizes sugar dispensers to manage mealybug infestations. The density of SDs tested in this study was shown to be relatively effective for mealybug management, reducing application costs in comparison with a previous study [36]. On the other hand, from our study, it is not possible to state if the tested density can be considered the optimal one.

Greater mealybug infestations were recorded in vineyards with a higher presence of the ant *L. paralienus*. *Lasius* ants are characterized by a high tendency to feed on hemipteran honeydew [37]. However, the method used to estimate ant presence, while providing a relative abundance estimation of ants, is not able to provide a consistent estimation of the local ant colony density. Considering the potential important role of ants in affecting mealybug infestation, it would be recommended to verify the ant assemblages in the Lambrusco vineyard areas of Emilia, particularly in sites mostly infested by mealybugs as evinced by recent faunistic studies in other vineyard cultivation areas of northern Italy [41].

The SD density tested in this experiment (80/ha) was shown to be in general effective in reducing mealybug infestations, providing a mean of a 22% decrease in bunches infested by mealybugs and a reduction in the mealybug density per bunch.

Sugar dispenser density tested in the present trial significantly increased the larval density of the predator *C. mountrouzieri*. Although predator larvae are not a direct measure of biological control intensity, this parameter should be an indication of a consequent augmentation of predation activity. This increase in predator larvae densities is likely correlated to reduced attending activity of ants and lower disturbance by these social insects against mealybug predators in vineyard plots with SDs. Similarly, spiders and lacewings were the most abundant predators observed in *Delottococcus aberiae* De Lotto (Hemiptera: Pseudococcidae) colonies in clementine mandarin orchards in Spain, and the exclusion of mutualistic ants with sticky barriers increased their abundance [42].

The tested SD density set in this experiment for reducing mealybug infestations showed a strong local effect on mealybug density reduction on bunches, denoting a decreased efficacy on plants sampled 5 m from SDs (0 m).

Although the effectiveness of the tested SD density on mealybug infestations was globally lower than that (39%) recorded with a greater SD density (120/ha) in a previous trial [7], the present study is a further validation of the general success of the method. Despite the medium-high infestation in the monitored vineyards, the harvest was carried out regularly; SD use could be considered an integrative method to mitigate mealybug infestation and enhance the efficacy of natural enemies, in particular the predator *C. mountrouzeri*.

The density of SDs tested here was not effective in enhancing parasitism. Indeed, parasitism by random estimation was not increased at the present dose of dispenser. Moreover, parasitism on colonies, which was higher on C plots, was likely driven by higher mealybug infestations in control plots; this should explain the apparent contradiction of why parasitism on colonies was higher in C than SD plots. This is in accordance with previous investigations on different multitrophic systems involving ants and parasitoids of honeydew producers or other pests, showing that at least in some circumstances, ants do not necessarily provide direct protection against (or interfere with) these natural enemies of pests [23,43,44].

The effectiveness of SDs in the treated plots showed a strong local effect of this method, demonstrated by the lower density of mealybug/bunch on the plants in which the artificial devices were set. In other words, a relative reduction in efficacy was noted on plants 5 m from a sugar dispenser, although the percentage of infested bunches did not reveal any difference. From this evidence, it can be deduced that dispenser density tested in this trial maintains an effect on the aggregation pattern of mealybugs, limiting the diffusion of colonies, although the ability to reduce the size of individual colonies is progressively reduced when moving away from the points in which dispensers are set.

The inclusion of substances able to increase the loyalty of ants toward sugary dispensers may significantly improve their performance and should be considered in future tests [28]. Also, it should be interesting to evaluate whether the use of sugar dispensers can increase the predation activity of ants against other vineyard pests as demonstrated by the literature and studies [20,24].

Currently, specific sugar dispensers for ant distraction are not on the market [24], strongly limiting the practical application of this method. Notwithstanding, a sugar provision could be obtained by modification of “liquid ant baits” that are commercialized by some companies, taking care not to add insecticide to the sugar liquid. In any case, ant management, including sugar dispensers or artificial nectaries, is currently strongly supported for mealybug integrated control, also in view of the interesting results of other experiments [11,22,25,27].

In conclusion, this work underlines and corroborates, as evinced by other studies, the importance of ants in affecting mealybug infestation, confirming the need to include management of these social insects in vineyard protection plans.

## Figures and Tables

**Table 1:** Vineyards involved in the experiment.

Site	Latitude	Longitude	Variety	Total Area of Farm	Plot Size (Control + Sugar Dispenser)
A	44.83491° N	10.72488° E	Lambrusco Marani	8 ha	0.52 ha
B	44.71957° N	10.61416° E	Ancellotta	6.6 ha	0.2855 ha
C	44.79276° N	10.79912° E	Lambrusco Salamino	11 ha	0.1225 ha
D	44.79636° N	10.82416° E	Ancellotta	9 ha	0.1872 ha

## Data Availability

The original contributions presented in this study are included in the article. Further inquiries can be directed to the corresponding author.
